# The Impact of Digital Transformation on Nursing Professional Experience: Critical Interpretive Synthesis Review Developing the Fragmented Professional Transition in Nursing (FPT-N) Model

**DOI:** 10.2196/94189

**Published:** 2026-08-25

**Authors:** Yan Sun, Shangqing Xu, Xiaoli Ma, Liyan Wang, Fenyan Wang, Tingting Su, Xueli Tao, Minjie Ma

**Affiliations:** 1 Department of Thoracic Surgery, The First Hospital of Lanzhou University (National Clinical Key Specialty), Donggang West Road 1#, Lanzhou, Gansu, 730000, China, 86 13893394458; 2 School of Nursing, Lanzhou University, Lanzhou, China; 3 Skills Training Center, The First Clinical Medical College of Lanzhou University, Lanzhou, China; 4 Gansu Provincial Thoracic Surgery Medical Quality Control Center, Lanzhou, China; 5 Gansu International Science, Lanzhou, China; 6 The First Clinical Medical College of Lanzhou University, Lanzhou, China

**Keywords:** digital transformation, nursing informatics, electronic health records, professional identity, critical interpretive synthesis, epistemic injustice, invisible work, workflow fragmentation, artificial intelligence, AI

## Abstract

**Background:**

Digital transformation through electronic health records (EHRs), telehealth, mobile health, and emerging AI has reshaped nursing work. Beyond questions of adoption and usability, nurses report disruptions to workflow continuity, professional identity, moral agency, and the meaning of care.

**Objective:**

This study aimed to synthesize interdisciplinary evidence on how digital transformation reshapes nursing professional experience and to develop the fragmented professional transition in nursing (FPT-N) model as a critical sociotechnical explanatory framework.

**Methods:**

A critical interpretive synthesis was conducted across nursing, health informatics, and organizational literature. A structured search of PubMed or MEDLINE, CINAHL, PsycInfo, and Scopus was followed by purposive theoretical sampling for conceptual richness. We included primary qualitative and mixed methods studies, 1 quantitative observational workflow study, and secondary syntheses used for triangulation. Qualitative evidence was appraised with the Critical Appraisal Skills Programme qualitative checklist to inform interpretive weighting. We used line-by-line coding, constant comparison, memoing, and synthesizing arguments.

**Results:**

The search identified 2847 records. After removing 865 duplicates, 1982 records were screened, 124 full-text reports were assessed, and 13 evidence sources were included. The synthesis generated 3 interacting mechanisms: workflow fragmentation, epistemic injustice in data-centric care, and expansion of invisible digital articulation work. Quantitative triangulation showed that 2871 interruption events were recorded across 145 observed shifts in 1 EHR workflow study, supporting the interpretation that digital work can intensify cognitive load. Contextual moderators, including governance, staffing, interoperability, and digital capital, shaped whether nurses experienced adaptive reintegration or alienation.

**Conclusions:**

Digital transformation may reconfigure important parts of nursing work around data production, potentially reducing the visibility of narrative clinical judgment and amplifying hidden coordination labor in some contexts. The FPT-N model offers a critical explanatory framework for examining when digital health implementation supports adaptive reintegration vs fragmented professional transition. The model identifies actionable targets for design and governance: aligning EHR workflows with nursing cognition, reducing interruptive documentation burden, recognizing invisible digital articulation work, preserving narrative clinical judgment, and involving nurses in sociotechnical decision-making.

## Introduction

### Background and Rationale

Digital transformation has become a defining feature of contemporary nursing practice. Electronic health records (EHRs), telehealth, mobile health applications, and emerging AI systems are now embedded in documentation, communication, coordination, and clinical decision-making [1]. Evidence syntheses show that information and communication technologies influence multiple domains of nursing care, including information access, documentation quality, time allocation, nurse autonomy, and nurse-patient interaction [2,3]. Recent evidence on documentation burden and EHR-related workload further suggests that digital tools can intensify rather than simply relieve nursing work [4,5].

The dominant implementation language around digital health often emphasizes adoption, efficiency, and standardization. These terms are important, but they do not fully capture nurses’ lived experience of work redesign. Digital systems may standardize data fields while weakening narrative continuity; they may accelerate information retrieval while increasing documentation time; and they may improve traceability while creating new forms of invisible coordination. This tension is especially relevant to nursing because care depends not only on task completion but also on relational judgment, embodied knowledge, and continuous sense-making across changing clinical situations.

### Why a Critical Interpretive Synthesis Was Needed

This review used critical interpretive synthesis (CIS) because the question was not only whether digital technologies are accepted or effective but how digital transformation reorganizes nursing knowledge, identity, and moral agency. CIS is appropriate when heterogeneous evidence must be interpreted to generate a conceptual explanation rather than aggregated into a single effect estimate [6]. We used PRISMA (Preferred Reporting Items for Systematic Reviews and Meta-Analyses) 2020 to transparently report structured search and screening procedures and ENTREQ (Enhancing Transparency in Reporting the Synthesis of Qualitative Research) to support reporting of qualitative and interpretive synthesis decisions [7,8].

Existing technology acceptance approaches help explain perceived usefulness and ease of use, but they often undertheorize the professional and epistemic consequences of digital work. This study therefore moves beyond the question of whether nurses use technology and asks how digital systems may reshape what counts as nursing work and nursing knowledge. The aim of this study was to synthesize interdisciplinary evidence on how digital transformation reshapes nursing professional experience and to develop the fragmented professional transition in nursing (FPT-N) model as a critical sociotechnical explanatory framework.

## Methods

### Design

We conducted a CIS following the methodology described by Dixon-Woods et al [6]. CIS combines systematic searching with iterative theoretical sampling. The goal is to develop a synthesizing argument that explains a phenomenon across heterogeneous bodies of evidence. In this review, the phenomenon was the effect of digital transformation on nursing professional experience. We used thematic synthesis techniques, including line-by-line coding and analytic theme development, as practical analytic tools within the broader CIS approach [9]. We report the structured search and selection stages using the PRISMA 2020 flow diagram for transparency. As CIS uses purposive theoretical sampling rather than exhaustive aggregation, certain PRISMA items that assume a fixed protocol (such as detailed reasons for excluding every full-text record at the conceptual sampling stage) are reported as not applicable; the rationale for the conceptual sampling decisions is documented in the audit trail (Multimedia Appendix 1).

### Information Sources and Search Strategy

We searched PubMed or MEDLINE, CINAHL, PsycInfo, and Scopus for English-language literature published from January 2015 to December 2024. The search combined nursing terms with digital technology terms and professional experience constructs. Example search concepts included nurs*, electronic health record, telehealth, digital health, mobile health, artificial intelligence, workflow, professional identity, experience, workload, qualitative, interview, focus group, observational, and review. The full database-specific search strings are provided in Multimedia Appendix 2.

### Eligibility Criteria

Sources were eligible if they examined nurses’ professional experience in relation to digital technologies in clinical practice. We included primary qualitative studies, mixed methods studies with substantial qualitative findings, 1 quantitative observational study that directly quantified EHR-related workflow interruption, and secondary syntheses used for conceptual triangulation. We excluded studies focused solely on students, nonnursing populations, non–health care technologies, or technologies not central to nursing work. For the purposive CIS stage, conceptual richness was operationalized using four criteria: (1) thick description of nurses’ digital work in real clinical settings; (2) explicit analysis of workflow disruption, workarounds, role strain, autonomy, communication, or identity tensions; (3) contribution of a transferable concept relevant to workflow fragmentation, epistemic authority, or invisible digital articulation work; and (4) sufficient proximity to practicing nurses’ experience. Sources did not need to meet all 4 criteria, but inclusion in the interpretive sample required at least one strong empirical or conceptual contribution to the developing synthesizing argument.

### Study Selection and Sampling

**Figure 1.**
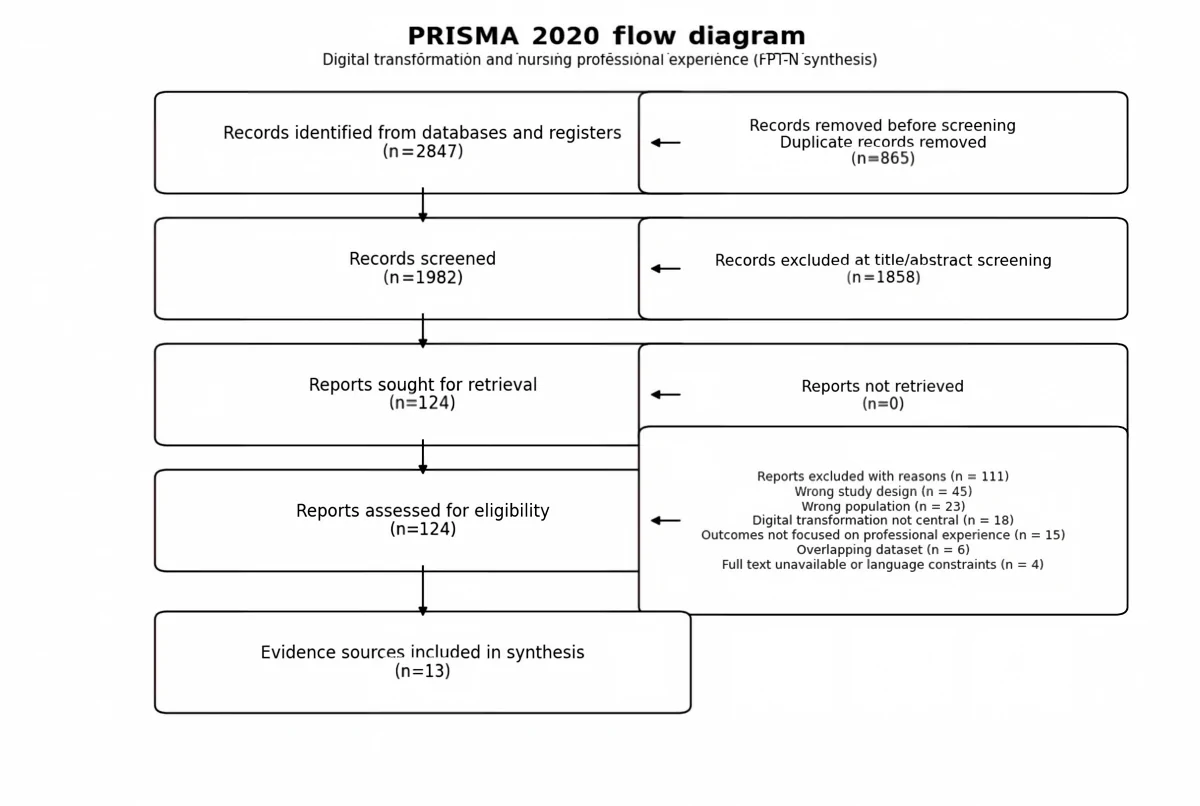
PRISMA 2020 flow diagram of evidence source identification, screening, eligibility assessment, and inclusion. FPT-N: fragmented professional transition in nursing.

The database search identified 2847 records. After removing 865 duplicates, 1982 records were screened by title and abstract. We assessed 124 full-text reports and included 13 evidence sources in the final synthesis (Figure 1). Two reviewers independently screened titles and abstracts, screened full texts, and resolved disagreements through discussion. During the CIS sampling stage, 1 reviewer led extraction and line-by-line coding, while a second reviewer checked inclusion rationales, construct allocation, and instances where an interpretive claim could exceed the immediate evidence base. Disagreements were resolved through discussion until consensus was reached. The structured search provided the initial sampling frame, and CIS purposive sampling was then used to identify sources with high conceptual relevance. Inclusion decisions were documented in a screening log and audit trail.

### Quality Appraisal and Interpretive Weighting

Primary qualitative studies were appraised using the Critical Appraisal Skills Programme (CASP) qualitative checklist [10]. In CIS, quality appraisal informs interpretive weighting rather than automatic exclusion. Studies with stronger methodological reporting were given greater weight when grounding empirical claims. Studies with narrower methods but high conceptual density were retained when they contributed to theory development, but their role was framed cautiously as conceptual rather than confirmatory evidence. For quantitative and secondary evidence, we assessed relevance, plausibility, and proximity to nurses’ original experience rather than applying the CASP qualitative checklist as a universal tool.

### Data Extraction, Synthesis, and Audit Trail

We extracted bibliographic information, evidence type, setting, sample or source composition, technology context, key findings, author interpretations, and relevance to FPT-N dimensions. Analysis proceeded through line-by-line coding, descriptive grouping, constant comparison, and memo-based development of analytic constructs [10]. To strengthen auditability, the audit matrix recorded (1) the empirical observation or review finding; (2) whether the source functioned as a primary empirical anchor or contextual or theoretical support; (3) the provisional FPT-N construct to which it contributed; (4) the memo rationale for retaining, merging, or revising the construct; and (5) whether the final statement was reported as an empirical finding or as a higher-order interpretation. The final synthesizing argument linked 3 constructs—workflow fragmentation, epistemic injustice, and invisible digital articulation work—into the FPT-N model. Analytic decisions, coding memos, inclusion rationales, and model development notes are summarized in Multimedia Appendix 1.

### Managing Secondary Evidence and Double Counting

As the evidence base included secondary syntheses, we explicitly managed the risk of interpretive layering and double counting. Secondary sources were used to map the broader field and triangulate concepts, not to duplicate primary-study quotations or inflate evidence volume. Where a primary study was also represented in a secondary review, the primary source was treated as the closer empirical anchor. This approach preserved the conceptual breadth of CIS while acknowledging the reduced proximity of secondary evidence to original nursing experience.

## Results

### Study Selection and Evidence Composition

The final synthesis included 13 evidence sources: primary qualitative or mixed methods studies, 1 quantitative observational study, and secondary syntheses that provided conceptual and contextual support. The included evidence covered EHR use, virtual care, telehealth, video consulting, digital competence, documentation burden, and workaround behavior. Table 1 summarizes the evidence type, setting or sample, main contribution, and FPT-N dimension supported by each source. These sources included a recent qualitative meta-synthesis of primary care digital health experiences [11]; a systematic review of digital competence [12]; an observational study of EHR interruptions [13]; and evidence on communication, workarounds, virtual care, and remote work [14-22].

**Table 1.** Characteristics of the included evidence sources and their contribution to the fragmented professional transition in nursing (FPT-N) model.

Evidence source	Evidence type	Setting or sample	Digital context	Key contribution	FPT-N dimension
Robles-Aguilar et al [11], 2025	Secondary	Primary care and qualitative meta-synthesis	Digital health	Adaptation and nurse-patient interaction	Professional transition and moderators
Krick et al [3], 2019	Secondary	Multiple care settings and 715 studies	Care technologies	Acceptance, effectiveness, and efficiency gaps	Efficiency illusion and implementation gap
Rouleau et al [2], 2017	Secondary	Overview of 22 systematic reviews	ICT^a^ in nursing care	Documentation, time, autonomy, and communication	Workflow fragmentation and nursing process
Wisner et al [5], 2019	Secondary	Integrative review of 18 studies	EHR^b^ cognitive work	Contextualization and synthesis of nursing information	Workflow fragmentation and epistemic injustice
Konttila et al [12], 2019	Secondary	Systematic review	Digital competence	Ethical, communication, and knowledge management domains	Contextual moderators and digital capital
Shan et al [13], 2023	Primary quantitative	Observational study and 145 shifts	EHR workflow interruption	Interruptions and multitasking increased mental workload	Workflow fragmentation
Blijleven et al [17], 2017	Primary qualitative	Hospital observations or interviews	EHR workarounds	Workflow misfit and workaround consequences	Invisible work and workflow fragmentation
Fraczkowski et al [18], 2020	Secondary	Integrative review	Nurse EHR workarounds	Workaround types and drivers	Invisible digital articulation
Bianchi and Ghirotto [19], 2022	Primary qualitative	Phenomenological interviews	Clinical workarounds	Unmet professional needs and moral tensions	Invisible work and moral agency
Forde-Johnston et al [14], 2023	Secondary	Integrative review	EHR and nurse-patient communication	Task-driven and closed communication patterns	Epistemic injustice and narrative loss
James et al [20], 2021	Secondary	NASSS^c^ systematic review	Video consulting scale-up	Hybrid care and coordination work	Moderators and digital articulation
Vaughan et al [21], 2024	Primary qualitative	Primary care nurses during COVID-19	Virtual care	Reshaped workflow, documentation, and autonomy	Professional transition and moderators
Anderson et al [22], 2024	Primary qualitative	General practice nursing and GenCo Study	Remote technology-mediated work	Transactional work and reduced decision involvement	Epistemic injustice and identity strain

a ICT: information and communications technology.

b EHR: electronic health record.

c NASSS: Nonadoption, Abandonment, Scale-up, Spread, and Sustainability.

To maintain alignment between evidence and interpretation, results were reported at 2 levels. First-order empirical findings refer to observations reported in included studies or reviews, such as documentation burden, interruptions, workarounds, and changes in virtual care communication. Higher-order interpretive claims refer to the CIS-derived explanation that these findings may interact to produce fragmented professional transition. We therefore use cautious wording, such as may, can, and suggests, when moving from evidence description to model-level interpretation.

### Theme 1: Workflow Fragmentation and the Efficiency Illusion

The first mechanism was workflow fragmentation. In the included evidence, digital systems could reorganize nurses’ work around documentation queues, alerts, authentication steps, and screen-based coordination. Secondary evidence showed that information and communications technology affects documentation time, information access, and communication [2,3]. EHR-specific syntheses described how documentation demands can shift attention away from situated clinical sense-making [5]. The quantitative observational study by Shan et al [13] recorded 2871 interruptions across 145 shifts, providing numerical triangulation for the interpretation that EHR-related work can increase cognitive load.

We define the efficiency illusion as the gap between promised technical efficiency and nurses’ experienced workload. The concept does not claim that digital tools are universally inefficient. Rather, it explains how apparent gains in retrieval, legibility, or standardization can be offset by hidden work, such as duplicated documentation, cross-system reconciliation, and repeated recovery from interruption. This definition responds directly to the need to clarify key concepts on first use.

### Theme 2: Epistemic Injustice in Data-Centric Care

The second mechanism was interpreted as epistemic injustice in data-centric care. Across the included evidence, structured digital systems could privilege codified, machine-readable data over embodied, narrative, and relational nursing knowledge. Evidence on EHR-mediated communication suggests that structured documentation can reduce the space for narrative interaction and create more task-driven communication patterns [14]. Digital competence literature also shows that training often focuses on technical operation, while ethical judgment, communication, and knowledge management capacities are equally important [12].

According to Fricker [15], epistemic injustice refers to wronging people in their capacity as knowers. In the nursing digital context, this concept helps interpret situations in which nurses’ experiential judgment is treated as secondary to data fields, templates, or algorithmic outputs. This is a higher-order interpretation built from empirical signals in the included evidence, rather than a direct empirical finding from any single study. We therefore frame erosion of epistemic authority as a possible mechanism in some digital implementation contexts, not as a universal consequence of digitalization.

### Theme 3: Expansion of Invisible Digital Articulation Work

The third mechanism was invisible digital articulation work. In the study by Allen [16], the sociology of nursing work shows that nurses perform extensive coordination labor that keeps health care systems functioning. In digital settings, this work can expand into data repair, cross-platform reconciliation, informal troubleshooting, workaround labor, and patient support for technology-mediated care. Evidence on EHR workarounds shows that nurses bypass or repair digital processes when system logic does not fit clinical contingencies [17,18]. Phenomenological evidence further suggests that workarounds may represent unmet professional needs and moral tensions rather than mere noncompliance [19].

We define invisible digital articulation work as the often-unrecognized work nurses perform to connect fragmented digital systems, clinical realities, and patient needs. Examples include reconciling information across platforms, documenting after the fact to match system requirements, mediating between remote and face-to-face care, and compensating for interoperability gaps. Recognizing this work is crucial because it determines whether digital implementation truly improves care or shifts costs onto nurses.

### Synthesizing Argument and the FPT-N Model

The FPT-N model integrates these 3 mechanisms into a critical sociotechnical explanation of how digital transformation may be experienced as fragmented professional transition. In this evidence base, digital transformation appeared to fragment nursing work when it interrupted workflow, narrowed the visible space for narrative judgment, and expanded hidden digital articulation labor. These mechanisms do not operate deterministically. Contextual moderators, including governance, staffing, interoperability, digital capital, and nurses’ involvement in technology decisions, shape whether digital transformation leads to adaptive reintegration or alienation. Evidence from virtual care and remote general practice nursing shows that technology-mediated work may become more transactional and may weaken nurses’ participation in decision-making if implementation is rapid and insufficiently participatory [20-22]. The model should therefore be read as a transferable explanatory heuristic that requires empirical testing in specific nursing contexts, rather than as a universally generalizable taxonomy.

## Discussion

### Principal Findings

This CIS found that digital transformation may reshape nursing professional experience through 3 interacting mechanisms: workflow fragmentation, epistemic injustice in data-centric care, and invisible digital articulation work. The main contribution of the FPT-N model is not the identification of any single burden, as prior studies have already described documentation burden, workarounds, and virtual care challenges. Its contribution is integrative and explanatory: it shows how these burdens may interact to produce a fragmented professional transition in which nurses renegotiate the meaning of care, their epistemic authority, and their role boundaries within digital systems. Recent work on digital technology and professional identity, documentation burden measurement, and health system documentation workload further supports the need to integrate professional identity and workload metrics in future implementation evaluations [23-25].

Recent nursing-focused evidence further confirms that technology may have mixed effects on workload, adoption, identity, implementation, and relational care, reinforcing the need for context-sensitive governance rather than efficiency-only evaluation [26-30].

### Novelty Relative to Existing Frameworks

Traditional technology acceptance models are useful for explaining perceived usefulness and ease of use, but they do not fully capture identity-level and epistemic consequences. General sociotechnical frameworks emphasize interactions among people, tools, tasks, and organizations, but they may not specifically theorize how nursing knowledge becomes less visible or how invisible digital articulation work expands. The FPT-N model adds value by framing digital transformation as a professional transition rather than only an implementation problem. This framing highlights that nurses are not simply users of technology; they are knowledge workers whose authority and moral agency can be strengthened or weakened by design and governance choices.

### Implications for Nursing Identity, Autonomy, and Moral Agency

Framing digital transformation as fragmented professional transition shifts attention from technology adoption alone to the professional consequences of digitally mediated care. Nursing identity is sustained through relational presence, narrative judgment, coordination, and accountability for continuity of care. When digital systems make documentation completion more visible than clinical sense-making, nurses may experience a mismatch between what the system measures and what they understand as good nursing. This mismatch can create identity strain, especially when nurses are expected to maintain relational and ethical care while absorbing additional documentation, troubleshooting, and cross-system reconciliation work.

Professional autonomy is also affected by how digital systems allocate authority. Decision support, standardized templates, and algorithm-mediated prompts can be useful when they extend nurses’ situational awareness and reduce preventable omissions. They become professionally problematic when they narrow the space for contextual judgment or require nurses to translate complex patient narratives into fields that do not capture uncertainty, relational cues, or moral priorities. The FPT-N model therefore emphasizes that moral agency should be treated as a matter of design and governance rather than solely as an individual competency.

### Data-Centric Care and Professional Governance

The wider implication of data-centric care is that nursing value may be inferred from what is easiest to capture, rather than from what is most important for safe and humane care. Documentation completeness, response time, and protocol adherence are legitimate metrics, but they do not fully represent anticipatory care, narrative interpretation, emotional support, or coordination across fragmented systems. A critical sociotechnical approach therefore requires governance structures that protect nurses’ epistemic contribution when data standards, EHR templates, and AI tools are introduced.

Practically, this means involving nurses in procurement, workflow redesign, implementation monitoring, and algorithm review; requiring usability assessments that include interruptions, narrative space, and hidden coordination work; and treating workaround patterns as diagnostic signals of sociotechnical misfit rather than as individual noncompliance. These governance mechanisms could help shift digital transformation from fragmentation toward adaptive reintegration.

### Contextual Moderators and Practice Implications

The moderators identified in the model specify practical levers for change. Governance can shift outcomes by involving nurses in procurement, workflow redesign, and algorithm review. Staffing models can recognize documentation, reconciliation, and patient technology support as real work rather than discretionary add-ons. Interoperability can reduce cross-system data repair, and digital capital can reduce inequity by ensuring that both nurses and patients have access to training and support. These actions operationalize the recommendation to recognize invisible digital articulation work through metrics, workload assessment, and participatory governance.

For health systems, recognizing invisible digital articulation work could involve time-motion audits, documentation burden dashboards, and structured reporting of workaround frequency. For technology developers, design evaluation should include nursing cognitive workflow and narrative clinical judgment, not only task completion time. For educators, digital competence should include ethical reasoning, epistemic humility, and communication in hybrid care, not only software operation.

### Boundary Conditions and Generalizability

The FPT-N model is intended as an explanatory framework for interpretation and further empirical testing. It should not be read as implying that all digital health implementations fragment nursing experience. The direction and magnitude of transition are likely to differ across clinical specialty, staffing model, digital maturity, interoperability, organizational culture, and the degree of nurse participation in design and governance. In settings where digital systems reduce duplication, preserve narrative communication, and make coordination labor visible, digital transformation may support professional reintegration rather than fragmentation.

### Strengths and Limitations

This review’s strengths include a transparent search process, explicit use of CIS, appraisal-informed interpretive weighting, and integration of primary, quantitative, and secondary evidence. The main limitation is that the final evidence base was relatively small and included several secondary sources. This composition increased conceptual reach but reduced proximity to original qualitative data in some areas. We addressed this by avoiding duplication of primary quotes from reviews, distinguishing empirical findings from higher-order interpretation, and treating secondary syntheses as contextual rather than confirmatory evidence. As CIS is designed for theory generation rather than prevalence estimation, the model should be considered provisional until tested in diverse nursing settings. Future empirical work should examine whether the proposed moderators predict adaptation, moral distress, turnover intention, perceived professional autonomy, or perceived epistemic authority.

### Conclusions

Digital transformation can improve information access and care coordination, but it may also fragment nursing professional experience when implementation privileges documentation logic over nursing cognition, marginalizes narrative clinical judgment, and expands unrecognized coordination work. The FPT-N model provides a critical explanatory framework for understanding these dynamics and for designing digital health implementation that protects nursing knowledge, reduces hidden labor, and supports adaptive professional reintegration. The broader significance of the synthesis is that digital nursing governance should evaluate not only whether systems are adopted but also whether they preserve professional identity, autonomy, moral agency, and nurses’ contribution as clinical knowers. Future research should operationalize the FPT-N mechanisms in empirical studies and test whether participatory governance, interoperability, and workload-sensitive design reduce professional fragmentation across nursing contexts.

## Supplementary material

10.2196/94189 Multimedia Appendix 1 Critical interpretive synthesis audit trail summary, including screening decisions, coding memos, and model development notes.

10.2196/94189 Multimedia Appendix 2 Full database search strategies.

10.2196/94189 Checklist 1 PRISMA 2020 checklist.

## Abbreviations

CASP: Critical Appraisal Skills Programme
CIS: critical interpretive synthesis
EHR: electronic health record
ENTREQ: Enhancing Transparency in Reporting the Synthesis of Qualitative Research
FPT-N: fragmented professional transition in nursing
PRISMA: Preferred Reporting Items for Systematic Reviews and Meta-Analyses
