# Supplementary material for: The Impact of Digital Transformation on Nursing Professional Experience: Critical Interpretive Synthesis Review Developing the Fragmented Professional Transition in Nursing (FPT-N) Model
Source: Asian Pac Isl Nurs J. 2026 Aug 25;10:e94189. doi: 10.2196/94189 (PMC13505599; doi:10.2196/94189)
Supplement: Supplementary file 1 [file apinj-v10-e94189-s001.docx]

# Multimedia Appendix 2. CIS Audit Trail Summary

## Purpose of the Audit Trail

This appendix summarizes the audit trail used to document the critical interpretive synthesis process, including screening decisions, conceptual-richness judgments, interpretive weighting, coding memos, and model development. The purpose was to make the interpretive pathway transparent without duplicating copyrighted full-text materials or qualitative quotations from secondary syntheses.

## Screening and Selection Log

Structured search records identified: 2,847. Duplicates removed: 865. Records screened: 1,982. Records excluded at title/abstract screening: 1,858. Full-text reports assessed: 124. Reports excluded with reasons: 111. Evidence sources included in final synthesis: 13.

Full-text exclusion reasons were grouped as follows: wrong study design, such as commentary/editorial or not empirical/synthesis (n = 45); wrong population, such as non-nurse sample or no nurse subgroup (n = 23); digital transformation not central (n = 18); outcomes not focused on professional experience, identity, or workflow (n = 15); overlapping or duplicate dataset with included evidence (n = 6); and full text unavailable or language constraints (n = 4).

## Operationalisation of Conceptual Richness

Conceptual richness was assessed using four criteria: (1) thick description of nurses' digital work in real clinical settings; (2) explicit analysis of workflow disruption, workarounds, role strain, autonomy, communication, or identity tensions; (3) contribution of a transferable construct relevant to workflow fragmentation, epistemic authority, or invisible digital articulation work; and (4) sufficient proximity to practicing nurses' experience. Sources did not need to meet all four criteria, but each retained source was required to contribute a strong empirical or conceptual insight to the developing synthesizing argument.

## Reviewer Roles and Decision Documentation

Two reviewers independently screened titles/abstracts and full texts and resolved disagreements by discussion. During the CIS sampling stage, one reviewer led extraction and line-by-line coding, while a second reviewer checked inclusion rationales, construct allocation, and claims that might exceed the immediate evidence base. Disagreements were resolved through discussion until consensus was reached.

For each included source, the audit matrix recorded bibliographic details, evidence type, digital technology context, key empirical findings, author interpretations, provisional FPT-N construct, interpretive weight, and whether the source served as a primary empirical anchor or contextual/theoretical support.

## Coding and Memo Development

Initial line-by-line coding focused on digital work organization, workflow interruption, documentation burden, communication changes, autonomy, professional identity, hidden coordination work, workarounds, and digital competence. Codes were then grouped through constant comparison into preliminary analytic categories.

Memoing was used to test whether early categories represented separate mechanisms or different manifestations of a shared mechanism. For example, documentation burden, alert fatigue, task switching, and authentication steps were grouped under workflow fragmentation. Workarounds, data repair, cross-system reconciliation, and informal troubleshooting were grouped under invisible digital articulation work.

## Distinguishing Empirical Findings From Higher-Order Interpretation

The final synthesis distinguished first-order empirical findings from higher-order interpretive claims. First-order findings included reported interruptions, documentation burden, workarounds, changes in nurse-patient interaction, virtual-care communication, and digital competence needs. Higher-order interpretation linked these findings with epistemic injustice and invisible work theory to develop the FPT-N model.

Because the model is theory-generating, broad claims were moderated in the revised manuscript using terms such as may, can, suggests, possible mechanism, and explanatory heuristic. The model is presented as requiring empirical testing in specific nursing contexts rather than as a universal taxonomy.

## Model Development

The synthesizing argument developed through iterative comparison of three mechanism clusters: workflow fragmentation, epistemic injustice in data-centric care, and invisible digital articulation work. Contextual moderators, including governance, staffing, interoperability, digital capital, training, and nurse participation in implementation decisions, were added when evidence suggested that the direction and magnitude of professional transition differed by organizational and technological context.

The final FPT-N model was refined to show interpretive pathways rather than deterministic causal effects. The revised figure and discussion emphasize boundary conditions and the possibility of adaptive reintegration when digital systems reduce duplication, preserve narrative judgment, and make coordination labor visible.
