# Supplementary material for: The Impact of Digital Transformation on Nursing Professional Experience: Critical Interpretive Synthesis Review Developing the Fragmented Professional Transition in Nursing (FPT-N) Model
Source: Asian Pac Isl Nurs J. 2026 Aug 25;10:e94189. doi: 10.2196/94189 (PMC13505599; doi:10.2196/94189)
Supplement: Supplementary file 2 [file apinj-v10-e94189-s002.docx]

# Multimedia Appendix 1. Full Database Search Strategies

## Scope and Limits

This appendix reports the structured search strategy used to identify the initial evidence base for the critical interpretive synthesis. The search covered English-language literature published from January 2015 through December 2024 and was supplemented by backward and forward citation chaining and purposive theoretical sampling for conceptual richness.

Databases searched: PubMed/MEDLINE, CINAHL, PsycINFO, and Scopus. Core concepts were nursing, digital technology, and professional experience/work organization. The final manuscript reports aggregate screening counts: records identified (n = 2,847), duplicates removed (n = 865), records screened (n = 1,982), full-text reports assessed (n = 124), and evidence sources included in the synthesis (n = 13).

## PubMed/MEDLINE

(("Nurses"[Mesh] OR nurs*[Title/Abstract]) AND ("Electronic Health Records"[Mesh] OR "electronic health record*"[Title/Abstract] OR EHR[Title/Abstract] OR "digital health"[Title/Abstract] OR telehealth[Title/Abstract] OR "mobile health"[Title/Abstract] OR mHealth[Title/Abstract] OR "Artificial Intelligence"[Mesh] OR "artificial intelligence"[Title/Abstract] OR algorithm*[Title/Abstract]) AND (workflow[Title/Abstract] OR workload[Title/Abstract] OR "professional identity"[Title/Abstract] OR experience*[Title/Abstract] OR autonomy[Title/Abstract] OR communication[Title/Abstract] OR "moral distress"[Title/Abstract] OR workaround*[Title/Abstract] OR "invisible work"[Title/Abstract]) AND (qualitative[Title/Abstract] OR interview*[Title/Abstract] OR "focus group*"[Title/Abstract] OR observational[Title/Abstract] OR review[Title/Abstract] OR "systematic review"[Title/Abstract])). Limits: English; publication dates from 2015/01/01 to 2024/12/31.

## CINAHL

(MH "Nurses+" OR TI nurs* OR AB nurs*) AND (MH "Electronic Health Records" OR TI "electronic health record*" OR AB "electronic health record*" OR TI EHR OR AB EHR OR TI "digital health" OR AB "digital health" OR TI telehealth OR AB telehealth OR TI mHealth OR AB mHealth OR TI "artificial intelligence" OR AB "artificial intelligence") AND (TI workflow OR AB workflow OR TI workload OR AB workload OR TI "professional identity" OR AB "professional identity" OR TI experience* OR AB experience* OR TI autonomy OR AB autonomy OR TI communication OR AB communication OR TI workaround* OR AB workaround*) AND (TI qualitative OR AB qualitative OR TI interview* OR AB interview* OR TI "focus group*" OR AB "focus group*" OR TI observational OR AB observational OR TI review OR AB review). Limits: English; 2015-2024.

## PsycINFO

(nurs*.ti,ab,id) AND ("electronic health record*".ti,ab,id OR EHR.ti,ab,id OR "digital health".ti,ab,id OR telehealth.ti,ab,id OR mHealth.ti,ab,id OR "artificial intelligence".ti,ab,id OR algorithm*.ti,ab,id) AND (workflow.ti,ab,id OR workload.ti,ab,id OR "professional identity".ti,ab,id OR experience*.ti,ab,id OR autonomy.ti,ab,id OR communication.ti,ab,id OR "moral distress".ti,ab,id OR workaround*.ti,ab,id) AND (qualitative.ti,ab,id OR interview*.ti,ab,id OR "focus group*".ti,ab,id OR observational.ti,ab,id OR review.ti,ab,id). Limits: English; 2015-2024.

## Scopus

TITLE-ABS-KEY(nurs* AND ("electronic health record*" OR EHR OR "digital health" OR telehealth OR mHealth OR "mobile health" OR "artificial intelligence" OR algorithm*) AND (workflow OR workload OR "professional identity" OR experience* OR autonomy OR communication OR "moral distress" OR workaround* OR "invisible work") AND (qualitative OR interview* OR "focus group*" OR observational OR review OR "systematic review")) AND PUBYEAR > 2014 AND PUBYEAR < 2025 AND (LIMIT-TO(LANGUAGE, "English")).

## Supplementary Searching and Purposive Sampling

Backward and forward citation chasing was used for conceptually relevant reviews and primary studies. During the CIS stage, additional sources were considered when they added conceptual density to the emerging synthesis, particularly for workflow fragmentation, epistemic authority, invisible articulation work, virtual care, and digital competence. Secondary syntheses were used to map concepts and context; duplicated primary-study material was not re-synthesized when overlap was identifiable.
