# Supplementary material for: The Impact of Digital Transformation on Nursing Professional Experience: Critical Interpretive Synthesis Review Developing the Fragmented Professional Transition in Nursing (FPT-N) Model
Source: Asian Pac Isl Nurs J. 2026 Aug 25;10:e94189. doi: 10.2196/94189 (PMC13505599; doi:10.2196/94189)
Supplement: Supplementary file 3 [file apinj-v10-e94189-s003.docx]

**PRISMA 2020 Checklist**

Manuscript #94189 | Asian/Pacific Island Nursing Journal

| **Title** | The Impact of Digital Transformation on Nursing Professional Experience: A Critical Interpretive Synthesis and the Fragmented Professional Transition (FPT-N) Model |
| --- | --- |
| **Article type** | Review / Evidence synthesis (Critical Interpretive Synthesis) |
| **Corresponding author** | Minjie Ma, MD |
| **Checklist source** | Page MJ, McKenzie JE, Bossuyt PM, et al. The PRISMA 2020 statement: an updated guideline for reporting systematic reviews. BMJ. 2021;372:n71. doi:10.1136/bmj.n71. |
| **Note** | This manuscript is a Critical Interpretive Synthesis (CIS). PRISMA 2020 was used to report the structured search, screening, evidence selection, and transparency items where applicable; ENTREQ also guided qualitative/interpretive synthesis reporting. Items related to meta-analysis, effect measures, and statistical synthesis are marked not applicable where appropriate. |

**Completed PRISMA 2020 checklist**

| **Section/Topic** | **Item** | **Checklist item** | **Location where item is reported** | **Response / Notes** |
| --- | --- | --- | --- | --- |
| Title | 1 | Identify the report as a systematic review. | Title page | Reported as a Review and titled as a Critical Interpretive Synthesis. The review uses PRISMA for transparent search/selection reporting and ENTREQ for qualitative/interpretive synthesis reporting. |
| Abstract | 2 | See the PRISMA 2020 for Abstracts checklist. | Abstract | Structured abstract reports Background, Objective, Methods, Results, and Conclusions, including databases searched, search/sampling approach, number of records and included evidence sources, core synthesis mechanisms, and model contribution. |
| Introduction | 3 | Describe the rationale for the review in the context of existing knowledge. | Introduction: Background and Rationale; Why a Critical Interpretive Synthesis Was Needed | The rationale explains why digital transformation may reshape nursing work beyond adoption/usability and why a CIS was needed to theorize workflow, knowledge, identity, and moral agency. |
| Introduction | 4 | Provide an explicit statement of the objective(s) or question(s) the review addresses. | End of Introduction | The manuscript states the objective to synthesize interdisciplinary evidence on digital transformation and nursing professional experience and to develop the FPT-N model. |
| Methods | 5 | Specify the inclusion and exclusion criteria for the review and how studies were grouped for the syntheses. | Methods: Eligibility Criteria; Study Selection and Sampling; Managing Secondary Evidence and Double-Counting | Eligibility criteria specify nurses' professional experience in relation to digital technologies in clinical practice, eligible source types, exclusions, and conceptual richness criteria for CIS sampling. Secondary syntheses are used for conceptual triangulation rather than duplicated primary evidence. |
| Methods | 6 | Specify all databases, registers, websites, organisations, reference lists and other sources searched or consulted to identify studies. Specify the date when each source was last searched or consulted. | Methods: Information Sources and Search Strategy; Multimedia Appendix 1 | The manuscript reports searches of PubMed/MEDLINE, CINAHL, PsycINFO, and Scopus for January 2015 to December 2024 and states that full database-specific search strings are provided in Multimedia Appendix 1. |
| Methods | 7 | Present the full search strategies for all databases, registers and websites, including any filters and limits used. | Methods: Information Sources and Search Strategy; Multimedia Appendix 1 | Reported in Multimedia Appendix 1 as database-specific strategies. The main text summarizes the nursing, digital technology, professional experience, qualitative, observational, and review concepts used. |
| Methods | 8 | Specify the methods used to decide whether a study met the inclusion criteria, including how many reviewers screened each record and report, whether they worked independently, and if applicable details of automation tools used. | Methods: Study Selection and Sampling | Two reviewers screened records independently and resolved disagreements through discussion. The structured search supplied the initial sampling frame, followed by CIS purposive sampling documented in the audit trail. No automation tools are reported as used for eligibility decisions. |
| Methods | 9 | Specify the methods used to collect data from reports, including how many reviewers collected data from each report, whether they worked independently, processes for obtaining or confirming data, and if applicable details of automation tools used. | Methods: Data Extraction, Synthesis, and Audit Trail | The manuscript reports extraction of bibliographic information, evidence type, setting, sample/source composition, technology context, key findings, author interpretations, and relevance to FPT-N dimensions. Extraction and interpretive decisions were supported by memoing and the audit trail. No automation tools are reported as used for data extraction. |
| Methods | 10a | List and define all outcomes for which data were sought. Specify whether all results compatible with each outcome domain in each study were sought. | Methods: Data Extraction, Synthesis, and Audit Trail | As a CIS, the review sought conceptual and empirical findings related to digital work organization, professional identity, knowledge practices, hidden labor, workflow fragmentation, epistemic authority, and invisible digital articulation work rather than predefined effect outcomes. |
| Methods | 10b | List and define all other variables for which data were sought. Describe any assumptions made about missing or unclear information. | Methods: Data Extraction, Synthesis, and Audit Trail; Table 1 | The extracted variables include source type, setting/sample, technology context, evidence contribution, and FPT-N relevance. NR is used where information was not reported or unavailable at screening/abstract level. |
| Methods | 11 | Specify the methods used to assess risk of bias in the included studies, including details of the tool(s) used, how many reviewers assessed each study and whether they worked independently, and if applicable details of automation tools used. | Methods: Quality Appraisal and Interpretive Weighting | Qualitative studies were appraised with the CASP Qualitative Checklist to inform interpretive weighting rather than automatic exclusion. Quantitative and secondary evidence were assessed by relevance, plausibility, and proximity to nurses' original experience. No automation tools are reported. |
| Methods | 12 | Specify for each outcome the effect measure(s) used in the synthesis or presentation of results. | Not applicable | No meta-analysis or effect-size synthesis was conducted. The review presents structured counts and conceptually oriented synthesis findings; the quantitative triangulation item reports interruption counts from one included observational study. |
| Methods | 13a | Describe the processes used to decide which studies were eligible for each synthesis. | Methods: Eligibility Criteria; Study Selection and Sampling; Data Extraction, Synthesis, and Audit Trail | Studies were selected for the CIS synthesis according to empirical relevance and conceptual richness, including thick descriptions of nursing work, workarounds, identity tensions, or theoretically informed interpretations relevant to workflow, epistemic authority, or invisible work. |
| Methods | 13b | Describe any methods required to prepare the data for presentation or synthesis. | Methods: Data Extraction, Synthesis, and Audit Trail | Data were prepared through extraction, line-by-line coding, descriptive grouping, constant comparison, and memo-based development of analytic constructs. |
| Methods | 13c | Describe any methods used to tabulate or visually display results of individual studies and syntheses. | Results: Table 1, Table 2, Table 3, Figure 2 | Table 1 summarizes evidence-source characteristics and FPT-N contributions. Table 2 maps mechanisms, evidence basis, and actionable implications. Table 3 presents quantitative triangulation evidence. Figure 2 presents the FPT-N model. |
| Methods | 13d | Describe any methods used to synthesize results and provide a rationale for the choice(s). | Methods: Design; Data Extraction, Synthesis, and Audit Trail | CIS was chosen for explanatory theory-building across heterogeneous evidence. Thematic synthesis techniques were used as practical analytic tools within the broader CIS approach. |
| Methods | 13e | Describe any methods used to explore possible causes of heterogeneity among study results. | Results: Synthesizing Argument and the FPT-N Model; Discussion: Contextual Moderators and Practice Implications; Boundary Conditions and Generalizability | Instead of statistical heterogeneity assessment, the synthesis interprets contextual moderators such as governance, staffing, interoperability, digital capital, and participatory implementation conditions. |
| Methods | 13f | Describe any sensitivity analyses conducted to assess robustness of the synthesized results. | Methods: Managing Secondary Evidence and Double-Counting; Discussion: Strengths and Limitations | Formal quantitative sensitivity analyses were not applicable. The review managed interpretive robustness by distinguishing primary evidence from secondary syntheses, avoiding double-counting, and distinguishing first-order findings from higher-order interpretation. |
| Methods | 14 | Describe any methods used to assess risk of bias due to missing results in a synthesis. | Not applicable; Discussion: Strengths and Limitations | Formal reporting-bias assessment was not performed because the review is a CIS and did not conduct effect-size synthesis. The limitations section acknowledges the small and synthesis-heavy evidence base. |
| Methods | 15 | Describe any methods used to assess certainty or confidence in the body of evidence for an outcome. | Methods: Quality Appraisal and Interpretive Weighting; Discussion: Strengths and Limitations | Formal GRADE/CERQual assessment was not conducted. Confidence was handled through appraisal-informed interpretive weighting, proximity to original nursing experience, and caution in distinguishing empirical findings from interpretive claims. |
| Results | 16a | Describe the results of the search and selection process, from the number of records identified in the search to the number of studies included in the review, ideally using a flow diagram. | Results: Study Selection and Evidence Composition; Figure 1 | The manuscript reports 2,847 records identified, 865 duplicates removed, 1,982 records screened, 124 full-text reports assessed, and 13 evidence sources included. Figure 1 presents the PRISMA 2020 flow diagram. |
| Results | 16b | Cite studies that might appear to meet the inclusion criteria, but which were excluded, and explain why they were excluded. | Figure 1; Multimedia Appendix 2 | Exclusion decisions and conceptual-sampling rationale are summarized in the PRISMA flow diagram and audit trail. Because the review is a CIS with purposive theoretical sampling, detailed citation of every excluded full-text report is not the purpose of the synthesis. |
| Results | 17 | Cite each included study and present its characteristics. | Results: Study Selection and Evidence Composition; Table 1; References | Each included evidence source is cited, and Table 1 summarizes evidence type, setting/sample, technology context, main contribution, and FPT-N dimension supported. |
| Results | 18 | Present assessments of risk of bias for each included study. | Methods: Quality Appraisal and Interpretive Weighting; Multimedia Appendix 2 | The manuscript states that CASP appraisal informed interpretive weighting rather than exclusion. Full per-study appraisal details are summarized in the audit trail rather than in the main text. |
| Results | 19 | For all outcomes, present, for each study: summary statistics for each group and an effect estimate and its precision where applicable. | Results: Theme 1; Table 3 | Not applicable to most included sources because this was an interpretive synthesis. The one quantitative observational workflow study is summarized with interruption events and observed shifts in Table 3. |
| Results | 20a | For each synthesis, briefly summarise the characteristics and risk of bias among contributing studies. | Results: Study Selection and Evidence Composition; Table 1; Methods: Quality Appraisal and Interpretive Weighting | The manuscript summarizes the composition of primary qualitative/mixed-methods studies, one quantitative observational study, and secondary syntheses. Quality appraisal is described as appraisal-informed interpretive weighting. |
| Results | 20b | Present results of all statistical syntheses conducted. | Not applicable | No statistical synthesis or meta-analysis was conducted. |
| Results | 20c | Present results of all investigations of possible causes of heterogeneity among study results. | Results: Synthesizing Argument and the FPT-N Model; Discussion: Contextual Moderators and Practice Implications | The synthesis interprets heterogeneity through contextual moderators rather than statistical subgroup analysis. |
| Results | 20d | Present results of all sensitivity analyses conducted to assess the robustness of the synthesized results. | Not applicable; Discussion: Strengths and Limitations | No quantitative sensitivity analysis was conducted. Robustness was addressed through interpretive weighting, double-counting safeguards, and caution about generalizability. |
| Results | 21 | Present assessments of risk of bias due to missing results for each synthesis assessed. | Not applicable | Not assessed because there was no effect-size synthesis and the review was a CIS. |
| Results | 22 | Present assessments of certainty or confidence in the body of evidence for each outcome assessed. | Methods: Quality Appraisal and Interpretive Weighting; Discussion: Strengths and Limitations | A formal certainty framework was not applied. The manuscript reports appraisal-informed interpretive weighting and limits the claims accordingly. |
| Discussion | 23a | Provide a general interpretation of the results in the context of other evidence. | Discussion: Principal Findings; Novelty Relative to Existing Frameworks | The discussion interprets the FPT-N mechanisms in relation to existing technology acceptance, socio-technical, professional identity, documentation burden, and nursing informatics literature. |
| Discussion | 23b | Discuss any limitations of the evidence included in the review. | Discussion: Strengths and Limitations | The manuscript discusses the relatively small final evidence base and inclusion of several secondary sources, which expanded conceptual reach but reduced proximity to original data in some areas. |
| Discussion | 23c | Discuss any limitations of the review processes used. | Discussion: Strengths and Limitations; Boundary Conditions and Generalizability | The manuscript acknowledges that CIS prioritizes theoretical relevance and concept development rather than exhaustive aggregation, and states that the FPT-N model requires further empirical testing. |
| Discussion | 23d | Discuss implications of the results for practice, policy, and future research. | Discussion: Implications for Nursing Identity, Autonomy, and Moral Agency; Data-Centric Care and Professional Governance; Contextual Moderators and Practice Implications; Conclusions | The manuscript discusses implications for procurement, workflow redesign, algorithm review, documentation-burden dashboards, usability assessments, professional governance, and future testing of the FPT-N model. |
| Other Information | 24a | Provide registration information for the review, including register name and registration number, or state that the review was not registered. | Abstract: Trial Registration; Methods/Discussion | The article states Trial Registration: Not applicable. The review was a CIS and was not presented as a registered intervention review. |
| Other Information | 24b | Indicate where the review protocol can be accessed, or state that a protocol was not prepared. | Methods: Data Extraction, Synthesis, and Audit Trail; Multimedia Appendix 2 | A formal protocol is not reported. The audit trail summary in Multimedia Appendix 2 documents screening decisions, coding memos, and model-development notes. |
| Other Information | 24c | Describe and explain any amendments to information provided at registration or in the protocol. | Not applicable | No registered protocol was reported; therefore protocol amendments are not applicable. |
| Other Information | 25 | Describe sources of financial or non-financial support for the review, and the role of the funders or sponsors in the review. | Submission metadata / author declarations | Funding/support information should be checked against the APINJ submission metadata and author declarations before production. No funding role is described in the manuscript text used for this checklist. |
| Other Information | 26 | Declare any competing interests of review authors. | Submission metadata / author declarations | Competing-interest declarations should be checked against the APINJ submission metadata and author declarations before production. No competing-interest statement is included in the manuscript text used for this checklist. |
| Other Information | 27 | Report which of the following are publicly available and where they can be found: template data collection forms; data extracted from included studies; data used for all analyses; analytic code; any other materials used in the review. | Multimedia Appendix 1; Multimedia Appendix 2 | Search strategies are provided in Multimedia Appendix 1. CIS audit-trail summary, screening decisions, coding memos, and model-development notes are provided in Multimedia Appendix 2. No analytic code was used for meta-analysis. |

*Prepared for production-stage supplementary upload in response to JMIR/APINJ request for a completed PRISMA checklist.*
